# Iron-Modified Biochar Reduces Phosphorus Leaching and Maintains Microbial Network Complexity in Acidic Soils Under Simulated Intense Rainfall

**DOI:** 10.3390/microorganisms14081715

**Published:** 2026-08-05

**Authors:** Yi Luo, Zihao Liu, Yongli Zhang, Chao Cui, Geqin Wang, Lili Dong, Shunli Wan

**Affiliations:** 1 College of Life and Environmental Science, Huangshan University, Huangshan 245041, China; luoyi@hsu.edu.cn (Y.L.);; 2 Anhui Academy of Agricultural Sciences Tea Research Institute, Hefei 230031, China; 3 College of Resource and Environment, Anhui Science and Technology University, Chuzhou 233100, China

**Keywords:** iron-modified biochar, phosphorus leaching, microbial network complexity, functional redundancy, Tax4Fun2

## Abstract

Although metal-modified biochar demonstrates high efficacy for phosphorus (P) removal in aqueous systems, its soil-scale mechanisms and ecological consequences under extreme rainfall remain largely unknown. In this study, we investigated how iron-modified biochar (BC+Fe) regulates P leaching and soil microbial communities in acidic soils using adsorption assays and column leaching experiments under simulated prolonged heavy rainfall. Mechanistically, BC+Fe exhibited adsorption kinetics that were better described by the pseudo-second-order model, consistent with a chemisorption-dominated P retention mechanism. Across six consecutive leaching events, BC+Fe significantly increased soil pH from 4.1 to 4.5 and reduced cumulative P loss by 37.7% compared to unmodified biochar (BC), with the most pronounced mitigation occurring during the initial leaching events when P losses were greatest. After leaching, soil total and available P concentrations under BC+Fe were approximately 3.4- and 3.7-fold higher, respectively, than under BC. Crucially, while both biochar types shifted bacterial community composition, BC+Fe maintained bacterial Shannon diversity and network complexity at levels comparable to the unamended soil and significantly higher than those under BC. Further analysis revealed that P leaching loss and soil pH were the primary environmental drivers shaping these microbial responses, and specifically, severe P loss was directly associated with simplified network complexity and intensified microbial competition (reflected by increased negative cohesion). Functional profiles inferred using Tax4Fun2 further showed that BC+Fe supported higher predicted microbial functional redundancy than both BC and the unamended control. Collectively, these findings demonstrate that iron-modified biochar mitigates P leaching through robust chemisorption and pH stabilization, while concurrently safeguarding microbial network complexity and functional redundancy. This dual benefit highlights the potential of iron-modified biochar as a sustainable amendment for maintaining soil ecosystem buffering capacity against severe hydrological stress.

## 1. Introduction

Acidic soils occupy approximately 30% (ca. 3.95 billion ha) of the global ice-free terrestrial surface and are widely distributed across major agricultural regions worldwide [1]. These soils are frequently constrained by declining fertility, which is closely associated with nutrient imbalances, the accumulation of potentially harmful microorganisms and metals, and, critically, the pronounced leaching losses of essential nutrients [2,3]. With the intensification of modern agriculture and its reliance on chemical fertilizers to sustain crop yields, acidic soils subjected to high-intensity or prolonged precipitation events are particularly vulnerable to nutrient depletion [4]. Under such conditions, nitrogen (N) and phosphorus (P) are readily mobilized and transported into adjacent aquatic systems, thereby exacerbating diffuse nutrient pollution and posing serious risks to aquatic ecosystem integrity and human health [5,6].

Among these nutrients, P is widely recognized as the primary driver of freshwater and coastal eutrophication, often exerting stronger control than nitrogen (N) over algal blooms and subsequent ecological degradation. Consequently, targeted P retention strategies are prioritized for effective eutrophication management [7,8]. Paradoxically, acidic soils are notorious for their strong P-fixing capacity, rapidly forming iron- (Fe) and aluminum- (Al) bound phosphates that render much of the added P unavailable to crops. Yet, despite this high fixation potential, substantial amounts of soluble P from repeated fertilization can still be rapidly leached via preferential flow paths during heavy rainfall, particularly in coarse-textured or structurally degraded soils [9]. Therefore, enhancing P retention against leaching-without exacerbating its irreversible fixation-constitutes a critical dual-benefit strategy. Such an approach can simultaneously improve agricultural P-use efficiency and mitigate downstream eutrophication risks.

In recent years, various approaches aimed at mitigating P leaching, such as polymer-based slow-release fertilizers, have been extensively explored [10,11]. Notably, biochar, a carbon-rich product of biomass pyrolysis, has emerged as a promising alternative for maintaining soil nutrient availability due to its tunable physicochemical properties and capacity to improve soil structure [12,13]. However, the direct application of pristine biochar presents notable limitations. In acidic soils, unmodified biochar typically exhibits a weak affinity for anions like phosphate. Consequently, rather than retaining P, it can paradoxically facilitate its downward migration through the soil profile [14]. This critical deficiency is primarily attributed to a limited anion-exchange capacity, insufficient active functional groups, and the potential disruption of soil aggregates under acidic conditions [15]. Beyond these intrinsic shortcomings, unmodified biochar may also undergo rapid surface oxidation and loss of labile organic fractions under intensive leaching conditions, particularly in acidic, coarse-textured soils [14,15]. Such aging processes can further diminish its already limited anion retention capacity and, in some cases, promote the downward transport of phosphorus via preferential flow paths [14]. To overcome these constraints, various surface modification strategies incorporating alkaline agents, macromolecular polymers, or metal elements have been developed to enhance biochar’s reactivity and selectivity [16,17,18]. Among these, iron (Fe) modification is particularly compelling, as the strong chemical affinity between Fe oxides or hydroxides and phosphate provides a robust mechanism for stable P retention in soils. The enhanced performance of iron-modified biochar is generally attributed to three complementary mechanisms [19,20]. First, iron oxides increase the density of reactive surface sites, thereby promoting phosphate adsorption through complexation. Second, their pH-buffering capacity alleviates acid-induced surface protonation and helps maintain phosphate sorption in acidic soils. Third, iron-bearing phases may partially protect the biochar matrix from oxidative aging, thereby sustaining its effectiveness during repeated leaching and wet–dry cycles.

Although iron-modified biochar has demonstrated high efficiency for P removal in aqueous systems, its efficacy for P retention in soils, particularly under intense or prolonged rainfall, remains insufficiently understood. Extreme precipitation events drive pronounced, pulsed nutrient losses, especially in regions with seasonal rainfall where nutrients accumulate during dry periods before being rapidly flushed into deeper soil profiles or water bodies [21,22]. Furthermore, the limited studies available tend to focus exclusively on physicochemical P retention, largely overlooking the cascading ecological consequences for soil microbial communities [23]. While recent findings indicate that modified biochars can influence soil enzyme activity and microbial structure by altering P fractions and soil pH [24], the specific effects of iron-modified biochar on microbial community assembly and functional resilience under intensive leaching scenarios remain a critical knowledge gap.

It has been well-documented that P availability is a key determinant of soil microbial community composition, interaction networks, and functional stability [25,26]. Adequate P supply promotes complex microbial co-occurrence networks and enhances community resilience to environmental disturbances, whereas abrupt P losses can destabilize these interactions and impair ecosystem functioning [27,28]. To mitigate such P losses, biochar amendments are increasingly utilized for P retention; however, the introduction of iron (Fe) to create iron-modified biochar introduces complex ecological ramifications beyond mere physicochemical retention. Because Fe redox cycling in acidic soils is intimately coupled with P mobilization, it serves as a critical driver of microbial community assembly. Consequently, iron-modified biochar acts as both a nutrient source and a microbial carrier, dynamically modulating the diversity and functional potential of soil bacterial communities. Given these microbial shifts, evaluating how biochar impacts the functional redundancy of these communities has been identified as a critical future research priority [29,30]. Yet, it remains unclear whether the P specifically immobilized by iron-modified biochar exerts regulatory effects on soil microbiota comparable to highly available P, particularly regarding microbial diversity, network complexity, and functional redundancy. Furthermore, microbial functional responses to biochar amendment under extreme rainfall remain poorly understood, despite the simultaneous intensification of P leaching and alteration of soil physicochemical conditions under such hydrological stress. This knowledge gap is particularly relevant in subtropical regions, where increasingly frequent and intense rainfall may disrupt P availability and microbial functional resilience. Addressing it is therefore essential for evaluating whether iron-modified biochar can sustain microbial functional redundancy while mitigating P loss, thereby supporting its environmental compatibility as a soil amendment.

In this study, iron-modified biochar was prepared and applied to acidic soils subjected to soil column leaching experiments under simulated prolonged heavy rainfall conditions and local fertilization practices. We hypothesized that enhanced P retention by iron-modified biochar, through Fe-associated adsorption and pH buffering, would alleviate microbial resource limitation under intensive leaching. This reduced resource stress was expected to weaken negative associations among taxa, thereby maintaining microbial network complexity and predicted functional redundancy and, ultimately, preserving the soil’s biological buffering capacity under hydrological disturbance. Specifically, the objectives of this study were to: (i) quantify the effectiveness of iron-modified biochar in reducing P leaching and regulating soil P availability under sustained heavy rainfall, and (ii) elucidate its impacts on soil bacterial community structure, network complexity, and functional redundancy in comparison with unmodified biochar and non-amended soils.

## 2. Materials and Methods

### 2.1. Iron-Modified Biochar Production

Iron-modified biochar (BC+Fe) was prepared using maize straw as the feedstock. The straw was cut into approximately 2 cm fragments, ground into powder, and sieved through a 40-mesh screen to ensure homogeneity. The processed material was divided into two equal portions. One portion (approximately 50 g) was immersed in a 1 mol L^−1^ FeSO_4_·7H_2_O solution at a solid-to-liquid ratio of 1:5 (*w*/*v*) and continuously stirred for 24 h at 25 °C to allow sufficient impregnation. The FeSO_4_·7H_2_O concentration of 1 mol L^−1^ was selected based on preliminary optimization experiments to ensure sufficient iron loading without compromising the structural integrity of the biochar matrix. Subsequently, the suspension pH was adjusted to 8.5 using 3 mol L^−1^ NaOH and stirred for an additional 24 h to promote in situ precipitation of iron oxides on the biomass surface. The solid fraction was then filtered and oven-dried at 60 °C overnight.

Pyrolysis was conducted in a muffle furnace under static-air conditions, rather than under an inert N_2_ atmosphere, at a heating rate of 10 °C min^−1^ to 400 °C, followed by a residence time of 2 h. Static-air pyrolysis was selected as a simple and cost-effective approach with potential scalability for agricultural applications. Compared with inert-atmosphere pyrolysis, limited oxygen exposure may promote partial surface oxidation and the formation or stabilization of oxygen-containing functional groups, such as carboxyl and hydroxyl groups, as well as reactive Fe oxide phases, thereby facilitating Fe anchoring and subsequent phosphate complexation. After cooling to room temperature, the biochar was repeatedly washed with deionized water until neutral pH was achieved and then dried to constant weight at 60 °C. SEM-EDS analysis of the representative BC+Fe sample showed an iron content of approximately 16.5% (*w*/*w*). The second portion of maize straw powder was pyrolyzed under identical conditions without chemical pretreatment and served as the unmodified biochar control (BC).

The P adsorption characteristics of BC and BC+Fe were evaluated through adsorption kinetic experiments, while surface morphology and elemental composition were examined using scanning electron microscopy coupled with energy-dispersive spectroscopy. The initial P concentration was set at 30 mg·L^−1^, the solid-to-liquid ratio was fixed at 0.01 g: 100 mL, and the suspension was shaken at 80 rpm at 25 °C. Supernatant aliquots were collected at 0, 5, 10, 30, 60, 120 and 240 min to analyze residual P. The obtained adsorption capacity data were fitted to pseudo-first-order and pseudo-second-order kinetic models to analyze the adsorption kinetic characteristics. SEM–EDS analyses were performed using an FEI Inspect F50 microscope (FEI Company, Hillsboro, OR, USA) equipped with a high-sensitivity XEDS system. Samples were mounted on metallic stubs with conductive carbon tape and sputter-coated with a 16 nm gold layer prior to observation.

### 2.2. Experimental Design and Sampling

Soil column leaching experiments were conducted under laboratory conditions at Huangshan University (29°43′ N, 118°19′ E), located in a subtropical monsoon climate zone with a mean annual temperature of 16.4 °C and an average annual precipitation of 1774 mm during 2018–2023. Red soils were collected from local fallow land at two depths: 0–20 cm (cultivated layer) and 20–35 cm (eluvial horizon). Following natural air-drying and sieving through a 2 mm sieve, the basic physicochemical properties of soils were determined. After gentle homogenization, soils were packed into transparent acrylic columns (15 cm inner diameter × 50 cm height) according to their natural stratification to maintain realistic vertical structure.

Three treatments were established in triplicate: (1) control without biochar or P fertilizer (CK); (2) pristine biochar with P fertilizer (BC); (3) iron-modified biochar with P fertilizer (BC+Fe). Each treatment was replicated three times. For the BC and BC+Fe treatments, biochar was applied at a rate of 22.5 t ha^−1^ [31] and mixed with the 0–20 cm soil layer. KH_2_PO_4_ was added to BC and BC+Fe at 351 kg ha^−1^ soil corresponding to the local agricultural practice, while CK received no P fertilizer.

Leaching was performed using deionized water to simulate prolonged heavy rainfall. Each leaching event delivered 2020.3 mL per column (equivalent to a rainfall depth of 114.3 mm). This rainfall intensity was selected to represent the average cumulative precipitation during the plum rain and summer storm periods in this region (2021–2025), based on data from the China Meteorological Data Service Centre (https://data.cma.cn/, accessed on 5 May 2026), and was applied at a simulated intensity of 5 mm h^−1^ over 24 h to mimic a typical heavy rainfall event. The leaching volume (V, mL) was determined by

V = Pr × A × 1000,(1)

where Pr is rainfall depth per event (mm) and A is column cross-sectional area (m^2^). A total of six leaching events were conducted at 7-day intervals, with a cumulative rainfall depth of 685.8 mm. This experimental design was established to simulate the natural rainfall pattern of alternating precipitation and dry periods typical of subtropical monsoon regions. Between successive leaching events, all soil columns were sealed and incubated at a constant temperature of 25 °C to maintain stable soil moisture conditions and allow P redistribution and fixation within the soil profile. Leachate was collected after each event for analysis. Post-experiment, 0–20 cm soil samples were collected via five-point composite sampling.

### 2.3. Analysis of P Retention and Leaching

Soil total phosphorus (TP) was determined using the perchloric acid–sulfuric acid digestion followed by the molybdenum–antimony–ascorbic acid colorimetric method, while available phosphorus (AP) was extracted using ammonium fluoride–hydrochloric acid and quantified by the same colorimetric approach [32]. P concentrations in leachate samples were measured using the potassium persulfate oxidation–ammonium molybdate spectrophotometric method [33]. The cumulative amount of leached P was calculated according to:

M = C × V(2)

where M represents the total mass of P leached (mg), C is the P concentration in the leachate (mg L^−1^), and V denotes the volume of leachate collected (L).

### 2.4. Soil Chemical Properties Analysis

Air-dried and finely ground soil samples were analyzed for basic chemical properties. Soil pH was measured using a calibrated pH meter at a soil-to-water ratio of 1:2.5. Total nitrogen was determined by the Kjeldahl digestion method, while available nitrogen was measured using the alkaline hydrolysis diffusion method. Total potassium was quantified after alkali fusion, and available potassium was extracted with 1.0 mol L^−1^ ammonium acetate (soil/solution ratio 1:5) and measured using a flame photometer. Prior to analysis, all extracts were filtered through Whatman Grade 40 quantitative filter paper (0.45 μm).

### 2.5. Soil DNA Extraction, Sequencing and Bioinformatic Analysis

Microbial genomic DNA was extracted from 0.50 g of fresh soil using the FastDNA Spin Extraction Kit (MP Biomedicals, Santa Ana, CA, USA) following the manufacturer’s protocol. DNA concentration and purity were assessed using a Qubit 3.0 Fluorometer (Thermo Fisher Scientific, Waltham, MA, USA). The V3–V4 region of the bacterial 16S rRNA gene was amplified using primers 341F and 805R. PCR amplification was performed with 2× Hieff Robust PCR Master Mix (Yeasen Biotechnology Co., Ltd., Shanghai, China) under a two-step cycling protocol to ensure amplification specificity and yield. Purified amplicons were obtained using a Qiagen Gel Extraction Kit (QIAGEN, Valencia, CA, USA) and sequenced on an Illumina MiSeq PE300 platform (Illumina, Inc., San Diego, CA, USA). Raw sequencing data are available in the NCBI database under accession number PRJNA1260197. Sequence processing followed a standardized bioinformatics pipeline (https://www.drive5.com/usearch/manual7/uparse_pipeline.html, accessed on 5 May 2026). Raw reads were quality-filtered using FASTP, merged using FLASH, and clustered into operational taxonomic units (OTUs) at 97% sequence similarity using UPARSE. Taxonomic assignment was performed with the RDP classifier against the SILVA 16S rRNA database. Finally, a total of 435,241 high-quality sequences were retained, ranging from 40,602 to 53,539 reads per sample. To avoid the bias in the downstream analyses, all samples were rarefied based on the minimum sequence depth, using the rarecurve function of ‘vegan’ package in R. The rarefaction curves for observed OTU richness approached asymptotic plateaus (Figure S1), indicating that the sequencing depth was sufficient to characterize the bacterial communities. Good’s coverage ranged from 0.995 to 0.999, further confirming adequate sampling completeness.

### 2.6. Statistical Analyses

All statistical analyses were performed using SPSS Statistics v24.0 (IBM, Armonk, NY, USA). Differences among treatments were assessed by one-way analysis of variance (ANOVA) with Tukey’s honestly significant difference (HSD) post-hoc testing (*p* < 0.05). Prior to ANOVA, data normality and homogeneity of variance were evaluated using the Shapiro–Wilk and Levene tests, respectively. Data were log-transformed when necessary to satisfy these assumptions. Principal coordinate analysis (PCoA) based on Bray–Curtis distance was conducted to explore differences in bacterial community composition. Significant differences among treatments were tested using permutational multivariate analysis of variance (PERMANOVA) with the ‘vegan’ package in R. Functional profiling of bacterial communities was predicted using the Tax4Fun2 v1.1.5 (R package), and the functional redundancy index (FRI) of each sample based on 16S rRNA gene sequence similarity was determined [34], which estimates the extent to which a given Kyoto Encyclopedia of Genes and Genomes (KEGG) function is supported by phylogenetically related taxa. Mantel tests were conducted to examine relationships between microbial traits (e.g., community composition and network complexity) and environmental variables, including soil properties and loss of available P, using the ‘linkET’ package in R. To control for multiple comparisons, *p*-values from the Mantel tests and Tax4Fun2-based functional comparisons were adjusted separately using the Benjamini–Hochberg false discovery rate procedure. Adjusted *p*-values (q < 0.05) were considered statistically significant. Finally, an exploratory structural equation model (SEM) was constructed using AMOS v24.0 (IBM SPSS Amos, IBM, Armonk, NY, USA) to evaluate the hypothesized direct and indirect relationships among BC+Fe application, soil pH, P leaching loss, absolute negative cohesion (|NegaCo|), and predicted functional redundancy. Before model fitting, the data were examined for linearity, normality, and severe multicollinearity. The model fitness was evaluated according to the following criteria [35]: a non-significant chi-square test (P > 0.05), χ^2^/df ≤ 2, root mean square error of approximation (RMSEA) < 0.06, comparative fit index (CFI) ≥ 0.95, and standardized root mean square residual (SRMR) < 0.08. Given the limited sample size, the SEM was interpreted as an exploratory assessment of the proposed pathways rather than definitive evidence of causality.

Co-occurrence networks were constructed to explore the bacterial interrelationships across treatments. Only OTUs with the relative abundances ≥ 0.01% and occurring in more than 4 samples were included. Pairwise Spearman correlations were computed, and robust correlations were defined as |r|> 0.75 and *p* < 0.01. Networks were visualized using the Fruchterman–Reingold layout in Gephi v0.11.2 (http://gephi.github.io/, accessed on 5 May 2026), and key topological properties including node number, edge number and average degree were extracted. Nodes were further classified into four categories based on within-module connectivity (Zi) and between-module connectivity (Pi): peripherals (Zi < 2.5 and Pi < 0.62), module hubs (Zi > 2.5), network hubs (Zi > 2.5 and Pi > 0.62) and connectors (Pi > 0.62). Only the network hubs, module hubs and connectors were considered as keystone species in ecological networks [36]. Community cohesion is a quantitative indicator of microbial interactions that reflects the overall degree of connectivity within a microbial community. This metric is calculated as the abundance-weighted sum of statistically significant positive and negative associations among taxa. Positive cohesion represents cooperative or co-occurring relationships, whereas negative cohesion reflects competitive or antagonistic interactions. Owing to its ability to capture emergent interaction patterns, cohesion analysis has been widely applied to characterize microbial associations across diverse ecosystems [37].

## 3. Results

### 3.1. Adsorption Performance of the Iron-Modified Biochar for P

**Figure 1:**
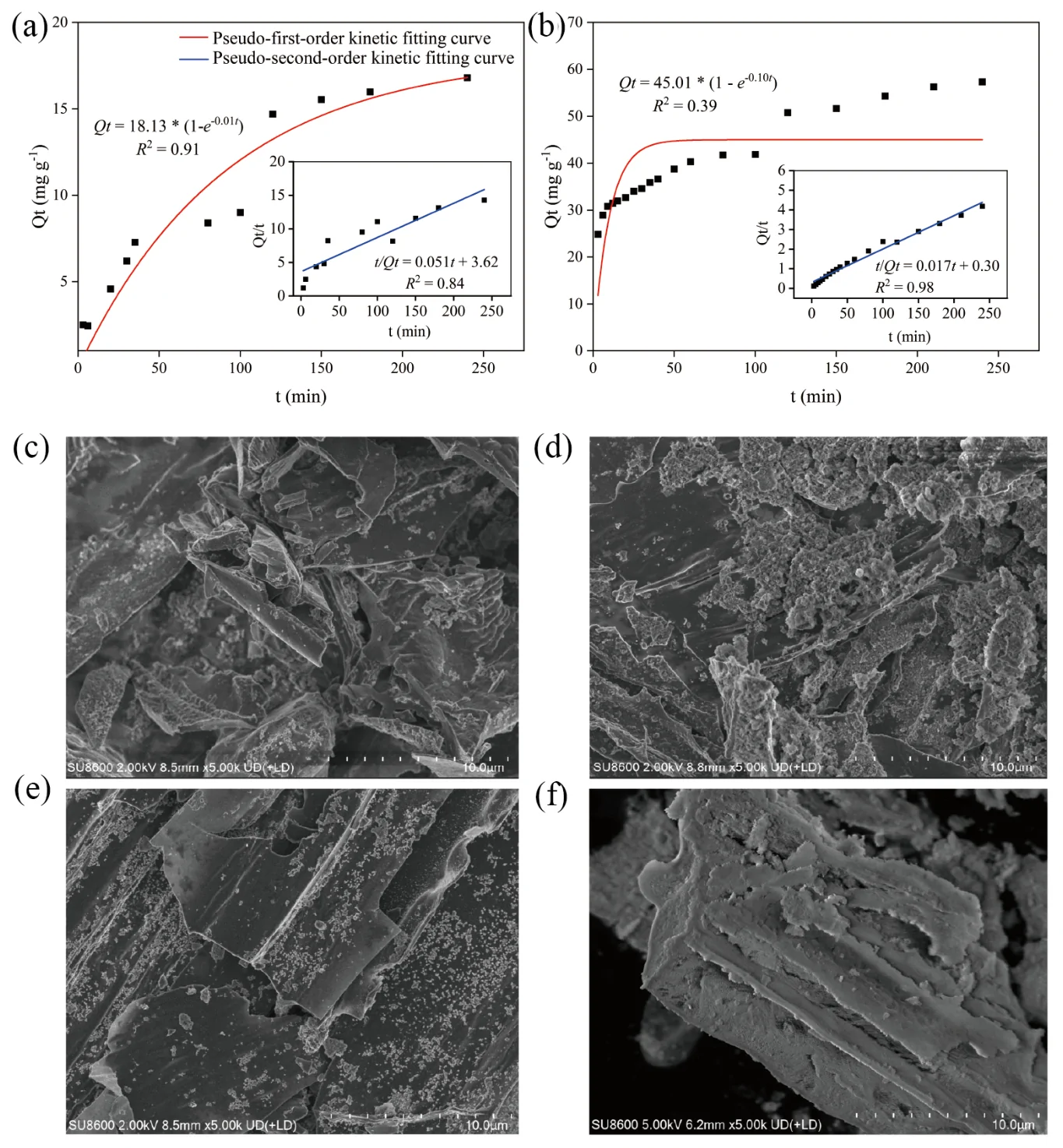
Kinetic fitting of unmodified biochar (**a**) and iron-modified biochar (**b**) for PO_4_-P adsorption. The adsorption reaction was performed at 25 °C with a phosphorus (P) concentration of 30 mg L^−1^, an initial pH of 7, and a rotating speed of 80 rpm. Scanning electron microscope (SEM) images of unmodified biochar (**c**), iron-modified biochar (**d**), unmodified biochar after PO_4_-P adsorption (**e**), and iron-modified biochar after PO_4_-P adsorption (**f**).

The P adsorption kinetics differed markedly between unmodified biochar (BC) and iron-modified biochar (BC+Fe). The adsorption data for BC were better described by the pseudo-first-order kinetic model (R^2^ = 0.91; Figure 1a), indicating that P removal was dominated by relatively simple, physically driven adsorption processes. In contrast, adsorption by BC+Fe closely followed the pseudo-second-order kinetic model (R^2^ = 0.98; Figure 1b), suggesting stronger and potentially more complex surface interactions than those observed for BC, although direct spectroscopic verification (e.g., FTIR or XPS) of the chemical state and bonding configuration of adsorbed P was beyond the scope of this study. 

Surface characterization further supported these kinetic differences. SEM images showed that BC exhibited a relatively smooth and homogeneous surface both before and after P adsorption (Figure 1c,e). In contrast, BC+Fe displayed a rougher surface with abundant crystal-like structures following modification and P adsorption (Figure 1d,f), indicating the formation of iron-containing active sites. SEM–EDS elemental mapping confirmed that iron was homogeneously and densely distributed on BC+Fe surfaces (Figure S2), providing a mechanistic basis for its enhanced and sustained P adsorption capacity.

### 3.2. P Leachate from Soils Across Different Treatments

**Figure 2:**
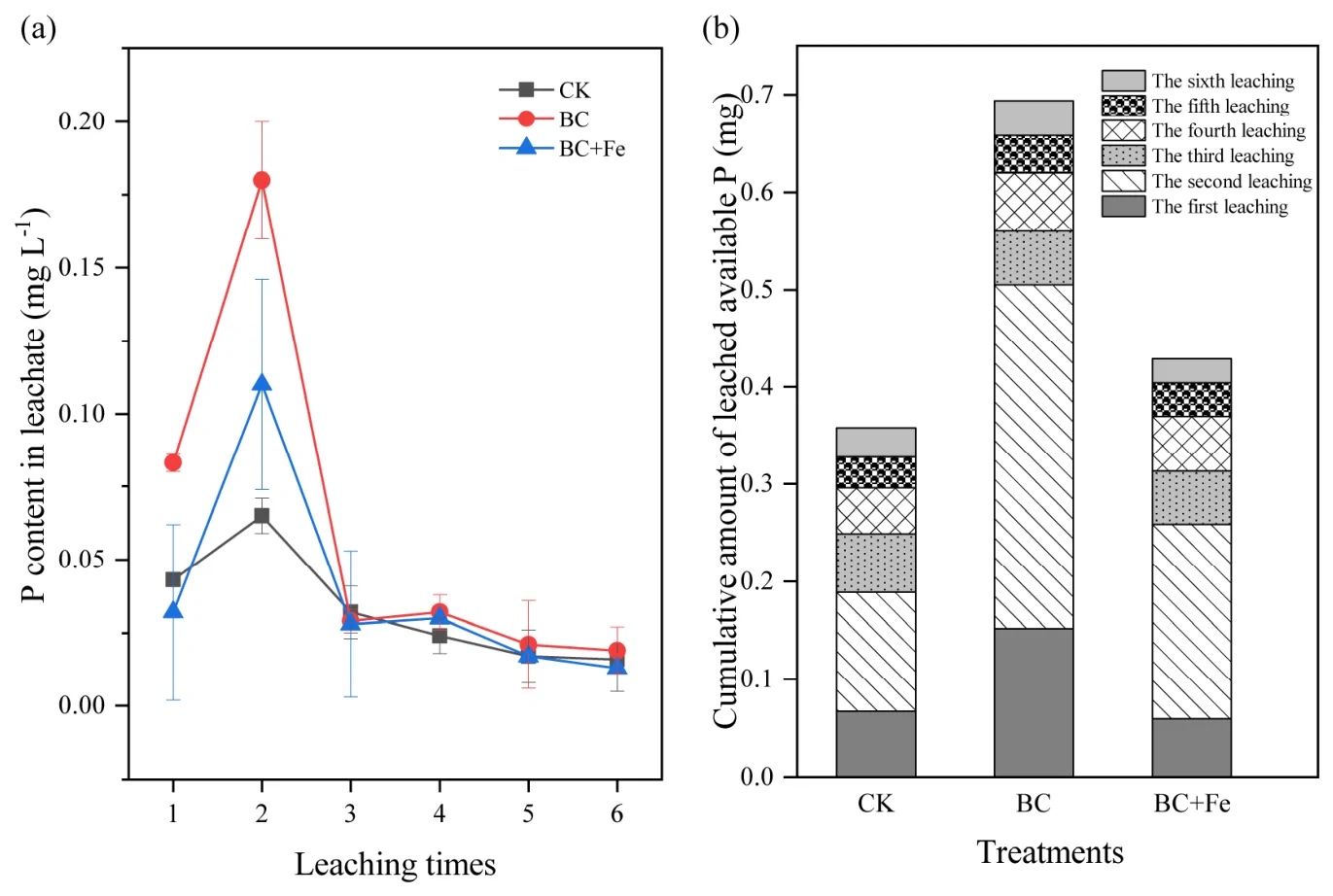
Changes in phosphorus (P) concentration in leachate (**a**) under six consecutive stimulated heavy rainfall events and the cumulative leaching of available P (**b**). Data are means with standard deviation (*n* = 3).

P concentrations in leachates were highest during the first two leaching events across all treatments (Figure 2a), likely due to the initially loose soil structure and enhanced preferential flow under simulated heavy rainfall. During this early stage, BC-amended soils exhibited substantially higher P concentrations in the leachate than BC+Fe soils, with BC showing a 1.59-fold increase relative to BC+Fe. The largest difference occurred during the second leaching event, when BC+Fe reduced leachate P concentrations by 38.9% compared to BC. Leachate P concentrations declined sharply with successive leaching events and eventually converged among treatments, indicating progressive depletion of mobile P pools. Notably, cumulative P leaching was greater under BC than under the unamended control, indicating that pristine biochar may promote, rather than mitigate, P leaching in acidic soils subjected to repeated heavy rainfall.

Consistent patterns were observed for cumulative P leaching (Figure 2b). More than half of the total leached P was lost during the first two leaching events, accounting for 53.9%, 72.8%, and 60.4% of total P loss in CK, BC, and BC+Fe treatments, respectively. After six leaching events, cumulative P loss from BC+Fe columns (0.43 ± 0.04 mg) was markedly lower than from BC columns (0.69 ± 0.06 mg), representing a 37.7% reduction compared with BC. Relative to the control (CK: 0.36 ± 0.04 mg), BC increased cumulative P loss by 0.33 mg (91.7%), whereas BC+Fe increased cumulative P loss by only 0.07 mg (19.4%). This modest increase relative to CK, combined with the substantial reduction relative to BC, underscores the effectiveness of iron modification in mitigating P leaching under repeated heavy rainfall.

### 3.3. Soil Chemical Properties Across Different Treatments

After six consecutive leaching events, significant differences in soil chemical properties were mainly observed for P-related parameters in the 0–20 cm soil layer (Table S1). Compared with BC, BC+Fe significantly increased soil TP and AP contents by 3.4-fold and 3.7-fold, respectively (*p* < 0.05), while exerting no significant effects on total or available nitrogen. Soil pH was significantly higher in BC+Fe and CK soils than in BC soils (*p* < 0.05), with lack of difference between BC+Fe and CK treatments. These results indicated that iron-modified biochar effectively retained P in the soil without substantially altering other nutrient pools.

### 3.4. Bacterial Community Structure and Predicted Functional Profiles

**Figure 3:**
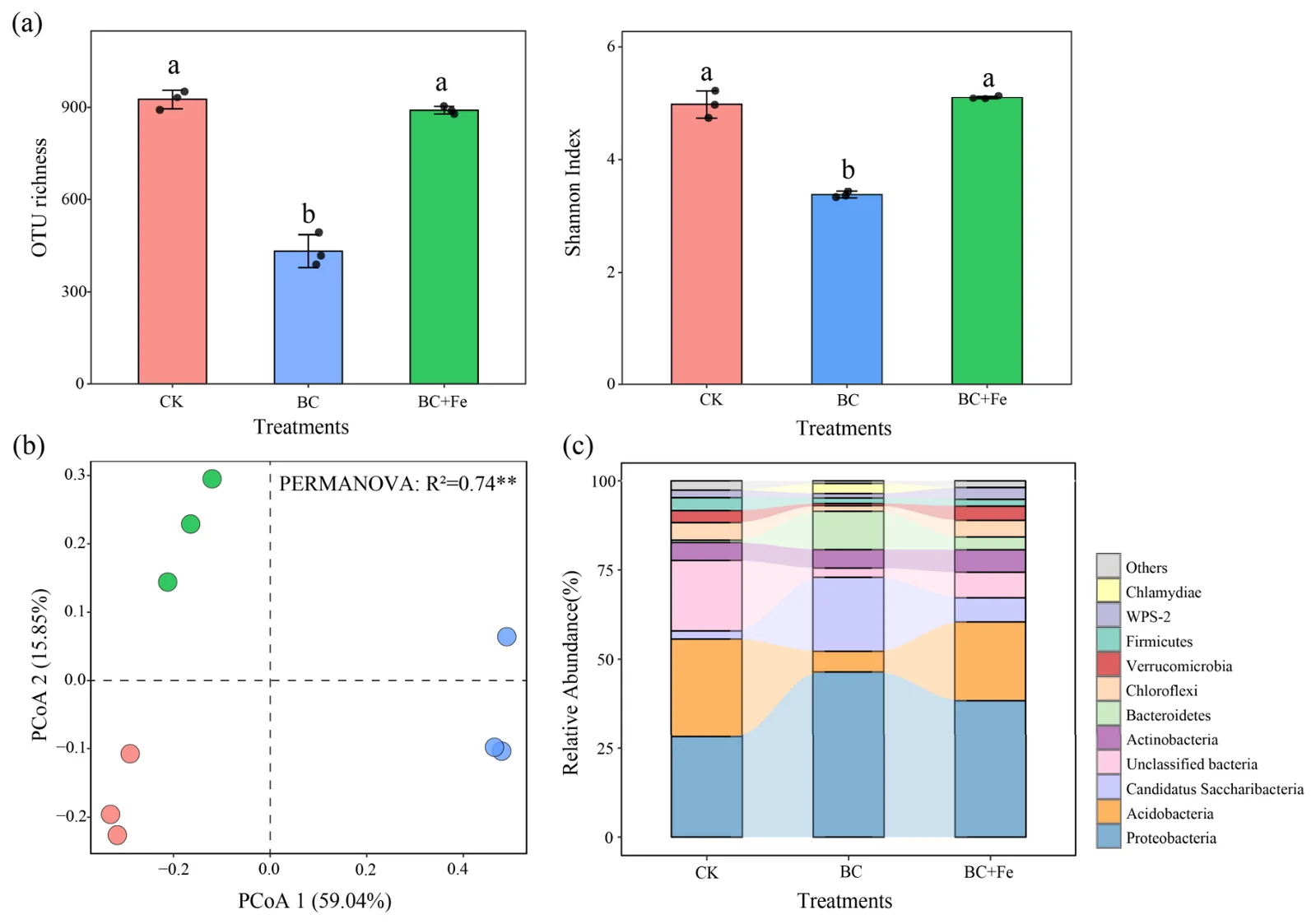
Bacterial α-diversity including OTU richness and Shannon diversity (**a**) and community composition (**b**). Bars are means with standard deviation (*n* = 3). Different letters indicate significant differences (*p* < 0.05) among soils. Relative abundances of bacterial communities at the phylum level (**c**). Others indicate these phyla with a relative abundance < 1%. **, *p* < 0.01.

Bacterial α-diversity, including OTU richness and Shannon diversity, was significantly higher in BC+Fe and CK soils than in BC soils (Figure 3a). Principal coordinate analysis (PCoA) revealed clear separation of bacterial community composition among treatments (Figure 3b), with differences confirmed by PERMANOVA (R^2^ = 0.74, *p* < 0.01). At the phylum level, Proteobacteria was the most abundant phylum across all treatments. Its relative abundance was significantly higher in BC soils than in CK, whereas BC+Fe showed no significant difference from CK. In contrast, Acidobacteria exhibited the opposite trend, with higher relative abundance in CK and BC+Fe soils than in BC (Figure S3). Other phyla, such as Actinobacteria and Bacteroidetes, remained comparatively stable (<10%) across all treatments.

**Figure 4:**
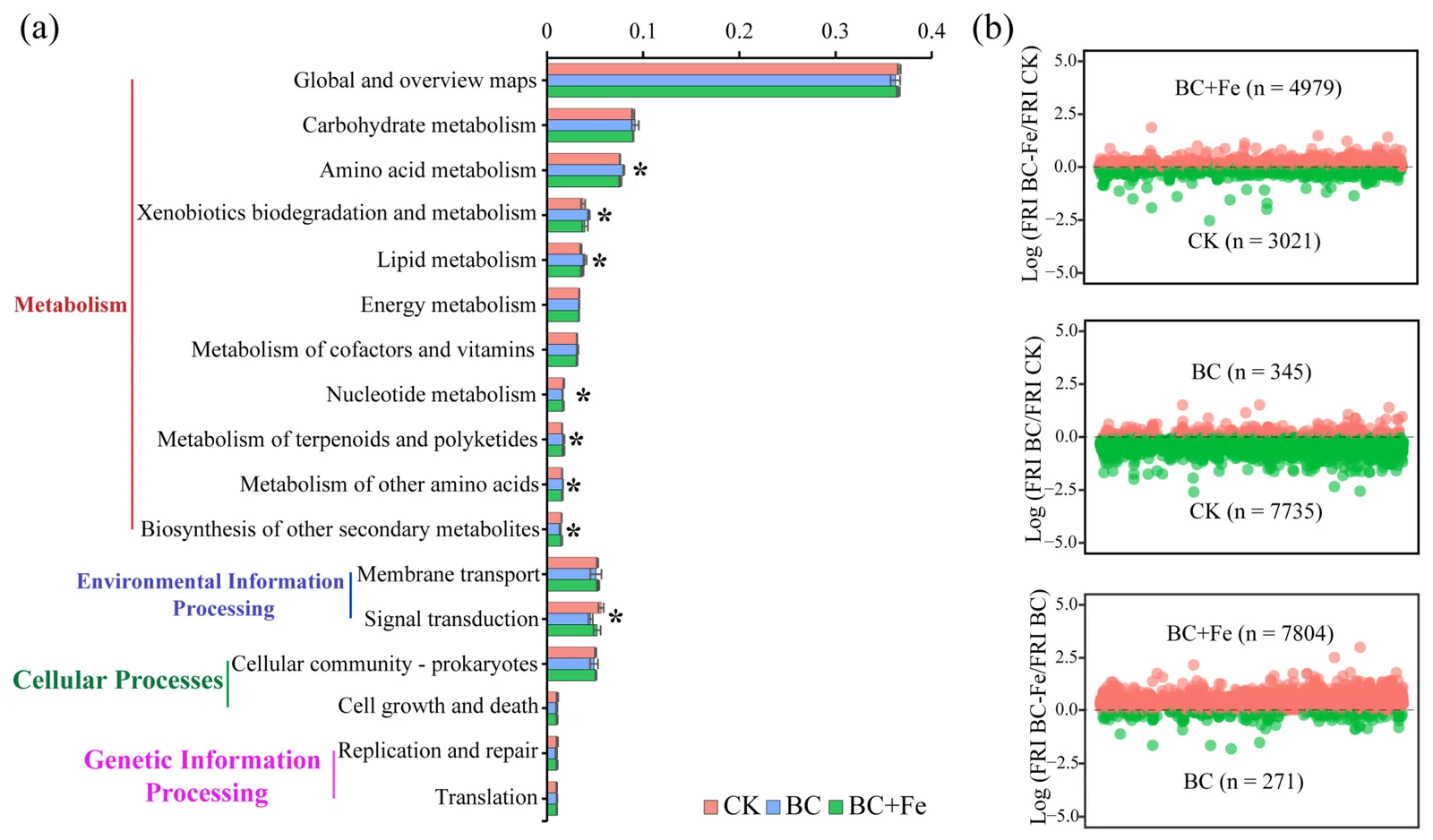
Metabolic pathways of bacterial communities in different soils predicted by Tax4Fun2 (**a**). Only relative abundance of metabolic pathways >1% are shown. Bars are means with standard deviation (*n* = 3). *, *p* < 0.05. Relative functional redundancy indices (FRIs) of bacterial communities in different soils (**b**). All predictions were based on a 97% 16S rRNA gene sequence similarity cut off. A log ratio greater than or less than 0 indicates that a function is more redundant in the corresponding soil.

Functional prediction based on Tax4Fun2 indicated that metabolism, environmental information processing, and cellular processes were the dominant predicted functional categories (Figure 4a). Eight of seventeen level-2 pathways differed significantly, primarily within metabolic pathways (7/17). Notably, bacterial communities in BC+Fe soils exhibited markedly higher predicted functional redundancy than those in BC and CK soils (Figure 4b). Specifically, 7804 and 4979 predicted functions showed higher redundancy in BC+Fe relative to BC and CK, respectively, whereas only 271 and 3021 functions exhibited higher redundancy in BC and CK compared with BC+Fe. These results suggest an enhanced predicted functional buffering capacity under iron-modified biochar application.

### 3.5. Co-Occurrence Network of Bacterial Communities

**Figure 5:**
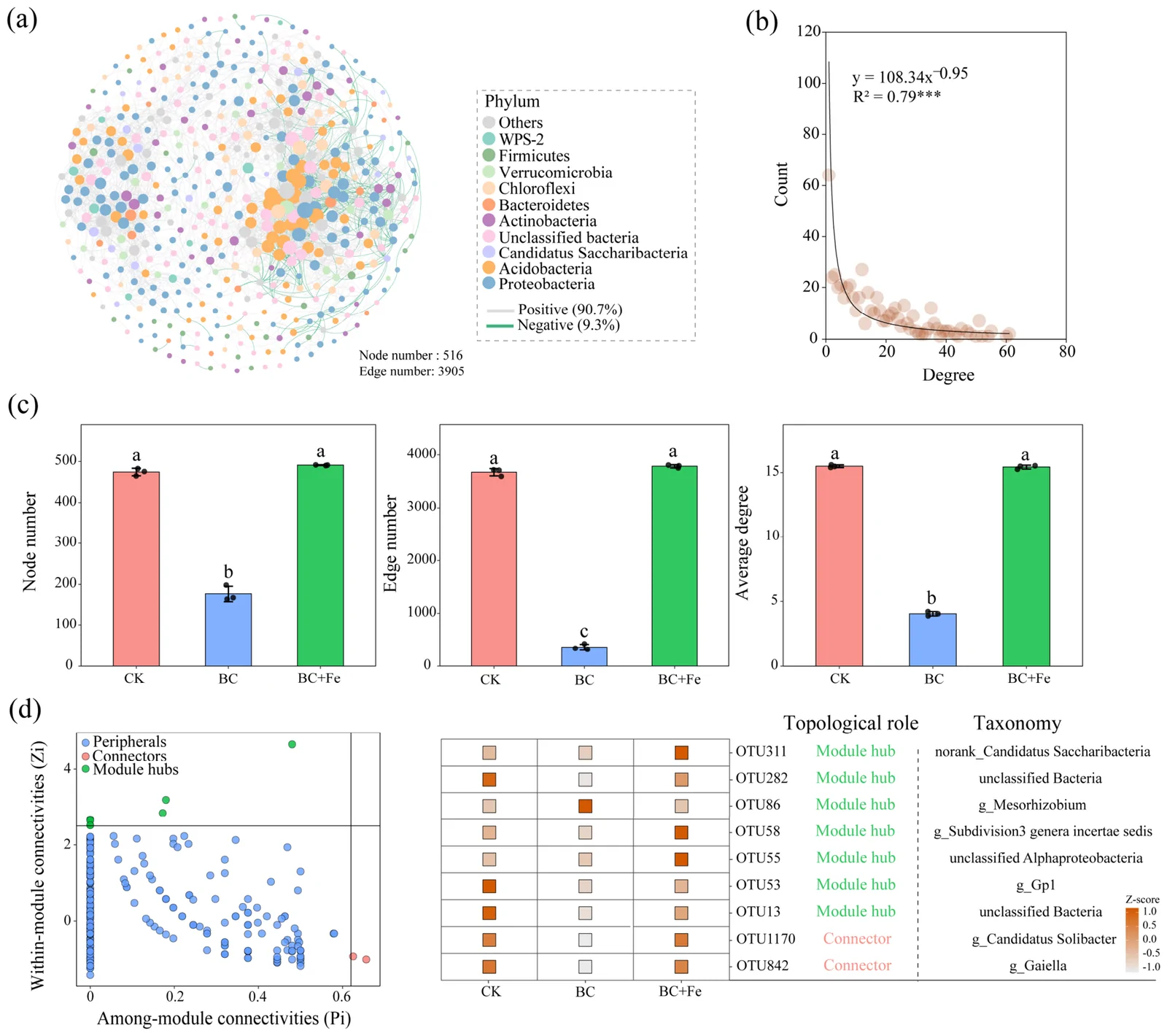
Co-occurrence network of bacterial communities (**a**) across soils and degree distribution pattern (**b**). Edges indicate significant interactions between nodes, with node size proportional to its degree. ***, *p* < 0.001. Comparison of key topological properties (**c**) including the node number, edge number and average degree. Bars are means with standard deviation (*n* = 3). Different letters indicate significant differences (*p* < 0.05) among soils. Zi-Pi plot (**d**) showing the classification of nodes to identify keystone species (left) and variation in their relative abundance (z-score transformed) across different soils (right).

Co-occurrence network analysis revealed pronounced differences in microbial interaction patterns among soils (Figure 5a). The overall network comprised 516 nodes and 3905 edges, with positive correlations accounting for 90.7% of all interactions, indicating predominantly cooperative relationships. The distribution of node number in both networks followed a power-law distribution (R^2^ = 0.79, *p* < 0.001), suggesting a scale-free and non-random distribution of network structure (Figure 5b). The node number (i.e., network size), edge number (connectivity), and average degree (network complexity) were significantly higher in CK and BC+Fe soils than in BC soils, with lack of differences between CK and BC+Fe soils (Figure 5c), suggesting greater network complexity and stability under BC+Fe application. Conversely, the absolute value of negative cohesion was significantly higher in BC soils than in CK and BC+Fe soils (Figure S4a). Positive cohesion peaked in CK soils among soils, with lack of difference between BC and BC+Fe soils (Figure S4b).

Nine keystone taxa were identified based on Zi–Pi plot, including seven module hubs and two connectors (Figure 5d). These keystone species were primarily affiliated with dominant phyla such as Candidatus Saccharibacteria, Proteobacteria, and Acidobacteria, suggesting their importance in maintaining network structure. Most keystone taxa exhibited higher relative abundances in CK and BC+Fe soils than in BC soils, with the exception of OTU86, which was more abundant in BC soils.

### 3.6. Relationships Between Key Microbial Traits and Soil Properties and P Leaching Loss

**Figure 6:**
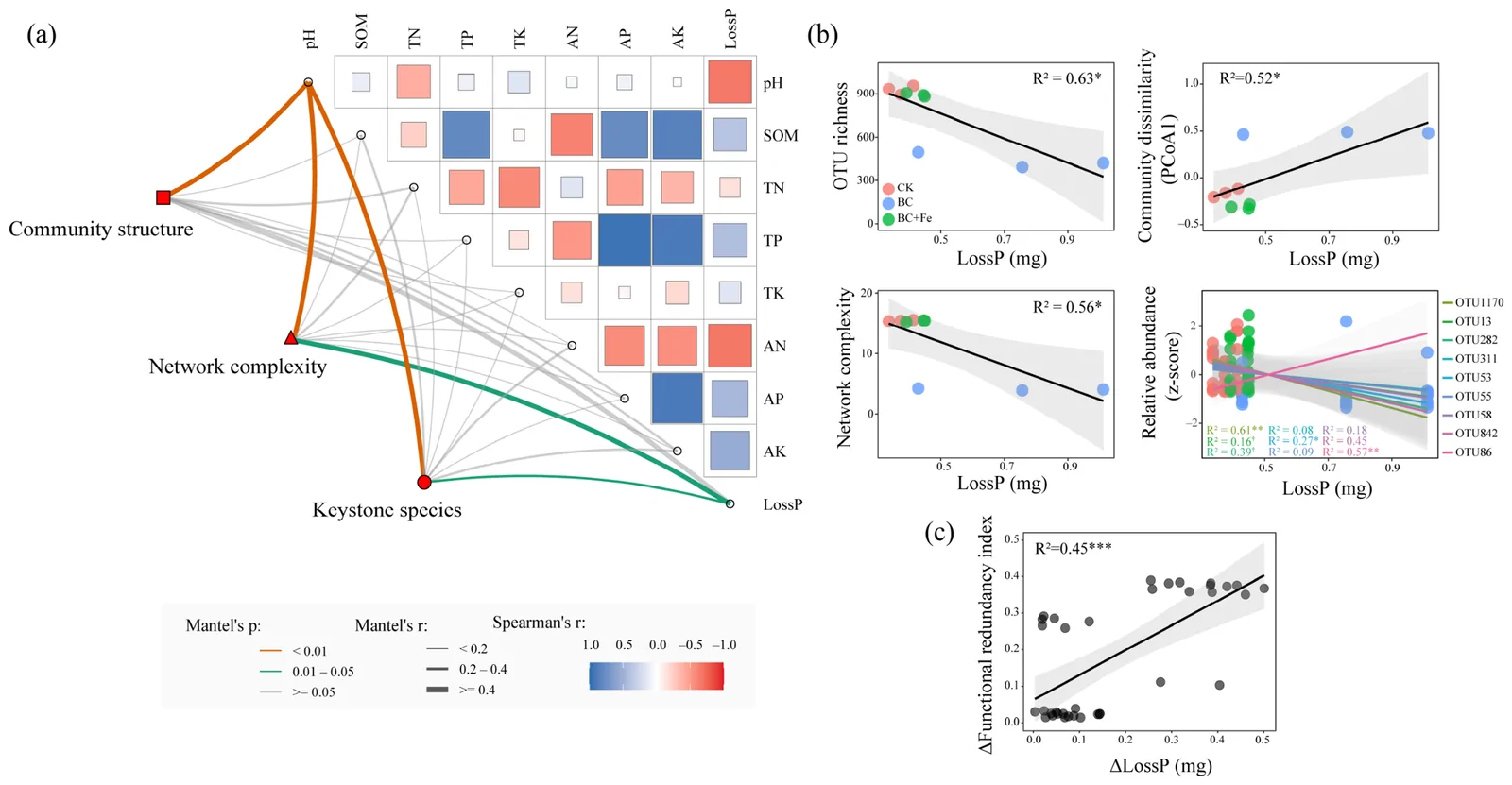
Potential drivers of bacterial community composition, network complexity and relative abundance of keystone species (**a**). Pairwise Spearman correlations of variables are represented by a color gradient. The edge width corresponds to Mantel’s r statistics, and edge color indicates statistical significance (999 permutations). Bacterial community composition and network complexity were represented by PCoA1 and average degree, respectively. LossP indicates the cumulative leaching loss of available P across six stimulated heavy rainfall events. Relationships between the cumulative leaching loss of available P and key microbial metrics including bacterial OTU richness, community structure (PCoA1), network complexity and relative abundance of each identified keystone species (**b**), as well as between changes in cumulative leaching of available P and functional redundancy of bacterial communities (**c**). Grey shaded areas indicate 95% confidence intervals of the regression fits. †, 0.05 < *p* < 0.1; *, *p* < 0.05; **, *p* < 0.01; ***, *p* < 0.001.

Mantel tests revealed significant correlations between soil pH and all examined microbial traits, as well as between P leaching loss (LossP) and network complexity and keystone species abundance (Figure 6a). Soil pH was negatively correlated with LossP, suggesting its indirect role in regulating P mobility through microbial-mediated processes. The absolute value of negative cohesion (|NegaCo|) decreased significantly with increasing soil pH but increased with rising LossP, whereas positive cohesion showed no consistent trend (Figure S5). Increasing LossP was associated with significant declines in OTU richness, network complexity, and the relative abundance of most keystone species (Figure 6b), whereas bacterial community dissimilarity and one keystone abundance (OTU86) increased. Functional redundancy index (FRI) of bacterial communities was also significantly correlated with LossP (Figure 6c), indicating that enhanced P retention under BC+Fe application was closely linked to more diverse, interconnected, and functionally redundant bacterial communities.

**Figure 7:**
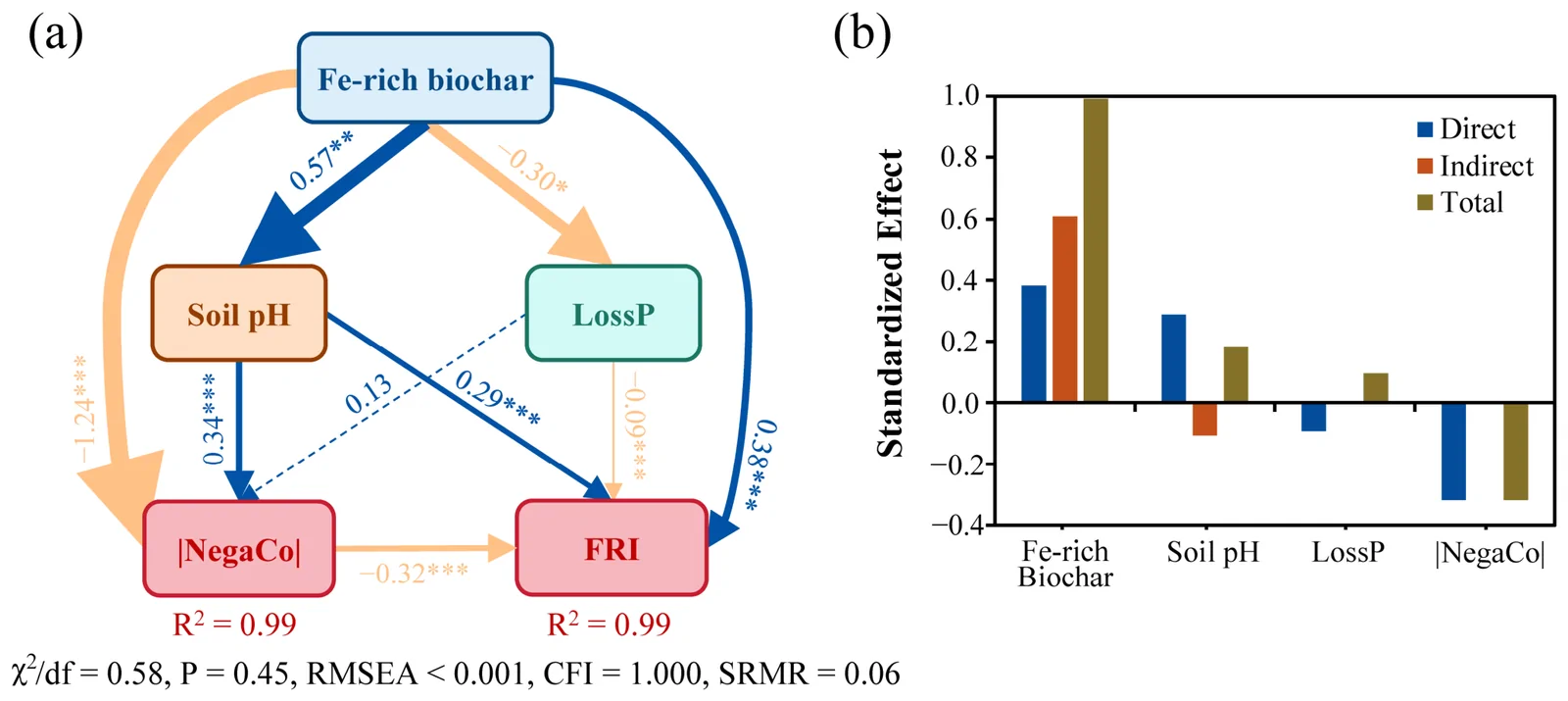
Using structural equation modelling to evaluate the potential pathways (**a**) and standardized effect sizes (**b**) of predictive factors for iron-modified biochar’s effects on predicted microbial functional redundancy. |NegaCo|, the absolute value of negative cohesion; FRI, the relative functional redundancy index of microbial communities that was represented by its PCA1 axis. *, *p* < 0.05; **, *p* < 0.01; ***, *p* < 0.001.

Structural equation modeling (SEM) was employed to disentangle the direct and indirect pathways through which biochar treatments influenced soil microbial functional redundancy (FRI) under the leaching disturbance regime. The model explained 57%, 30%, and 99% of the variances in soil pH, P leaching loss (LossP), and FRI, respectively, indicating good explanatory power (Figure 7a). Soil pH exerted the strongest direct effect on LossP (standardized path coefficient = –1.24). LossP directly promoted negative cohesion (standardized coefficient = 0.29), which in turn suppressed FRI (standardized coefficient = −0.32). Soil pH also exhibited a positive direct effect on FRI (0.34). The total indirect effect of BC+Fe on FRI (0.99) far exceeded its direct effect (–0.32), indicating that pH-mediated pathways dominated the overall effect. Among all predictors, |NegaCo| had the strongest standardized direct and total effects on FRI, whereas soil pH contributed the largest indirect effect (Figure 7b).

## 4. Discussion

### 4.1. Iron-Modified Biochar Efficiently Mitigates P Leaching Losses from Soil Under Prolonged Heavy Rainfall

A critical, albeit counterintuitive, finding of this study is that conventional biochar (BC) co-applied with phosphate fertilizer exacerbated P leaching from acidic soils under prolonged heavy rainfall. Rather than invalidating the biochar approach, this adverse effect compellingly justifies the necessity of iron modification. It is precisely this performance gap that underscores the functional significance of the iron-modified biochar (BC+Fe), which effectively transformed the amendment from a facilitator of P-release into a robust P-retention barrier. This stark contrast originates from fundamental differences in their underlying retention mechanisms. Unmodified biochar, with its macroporous structure and limited anion exchange capacity, likely enhanced preferential flow and anion mobility in the acidic soil profile, a mechanism that is consistent with previous reports [38]. The lower P loss under BC+Fe, together with its pseudo-second-order adsorption kinetics and Fe-rich surface characteristics, is consistent with enhanced P retention through Fe-associated chemisorption. However, because Fe-P bonding configurations were not directly characterized, this mechanism should be regarded as a plausible interpretation supported by the adsorption results and previous studies [39,40].

Consequently, this mitigated leaching directly accounts for the significantly higher residual total P (TP) and available P (AP) observed in the BC+Fe treatment (Table S1). From a mass balance perspective-given comparable initial soil TP and uniform fertilizer inputs across treatments-the substantial reduction in P leaching inherently translated into greater P retention within the soil solid phase. Furthermore, any intrinsic P introduced from the original biomass feedstock was efficiently retained by these same iron-driven immobilization mechanisms rather than being flushed away.

The divergent pH responses between treatments further elucidate the multi-faceted benefits of iron modification. Under intense leaching, soil pH in the BC treatment increased slightly to ~4.0, whereas the BC+Fe treatment buffered the soil pH, elevating it to a more favorable ~4.5. The contrasting pH responses may partly reflect differences in biochar aging under repeated leaching. Intensive wetting and drainage can remove soluble organic fractions, ash, and alkaline cations from pristine biochar, exposing oxidation-prone carbon surfaces to water and oxygen [41]. Subsequent surface oxidation may generate acidic oxygen-containing functional groups, thereby weakening the initial liming capacity of BC and contributing to soil acidification. In contrast, the Fe-bearing coating may partially protect the carbon matrix from direct oxidation and provide additional mineral buffering [41]. Crucially, this pH-buffering effect synergized with the retention mechanisms to alter P fractions. The moderate elevation of pH to ~4.5 under BC+Fe likely triggered the mobilization of a fraction of previously fixed Fe/Al–bound P into bioavailable pools [42,43]. This dual mechanism explains why BC+Fe supported both higher TP (through arrested leaching) and higher AP (through pH-driven mobilization) than BC under identical fertilization. Together, these results demonstrate that iron-modified biochar not only physically intercepts acute P leaching during extreme precipitation but also chemically optimizes the bioavailability of the retained P.

### 4.2. Iron-Modified Biochar Application Enhances Microbial Diversity and Network Complexity

Beyond P retention, iron modification effectively buffered the ecological disturbances induced by pristine biochar. While previous studies establish that conventional biochar application significantly alters bacterial taxonomic distribution [44,45], our ordination and diversity analyses revealed that communities under the BC+Fe treatment more closely resembled the unamended control (CK) than those under unmodified BC. Rather than amplifying the biochar-induced shifts, iron modification mitigated these disruptive effects, fostering a more stable and diverse microbial assemblage. At the taxonomic level, these dynamics were clearly reflected in the balance between copiotrophic and oligotrophic phyla. For instance, Proteobacteria were broadly enriched under both biochar treatments, likely reflecting their copiotrophic lifestyle and rapid exploitation of labile nutrients via phosphatase production [46]. Conversely, Acidobacteria, typically associated with oligotrophic conditions and the efficient utilization of recalcitrant P sources [47], were suppressed by conventional BC but maintained higher relative abundances in both CK and BC+Fe soils. This persistence suggests that iron modification moderated the acute nutrient imbalances caused by pristine biochar, preserving crucial niches for oligotrophic taxa [48].

To elucidate the ecological implications of these taxonomic shifts, we evaluated the relative abundances of predicted bacterial functional pathways. A key finding was the significant enrichment of environmental information processing pathways particularly those involved in signal transduction under the BC+Fe treatment compared to conventional BC (Figure 4a). These pathways are critical for coordinating microbial responses to environmental stress and nutrient fluctuations [49,50,51]. This enrichment suggests that iron-modified biochar promotes tighter intercellular regulation and cooperative resource allocation rather than indiscriminate metabolic expansion. Such a coordinated functional shift aligns perfectly with the increased microbial network complexity observed under BC+Fe [52]. By enhancing signaling capacity, BC+Fe likely facilitates more efficient interspecies cooperation, thereby supporting predicted higher functional redundancy without excessive metabolic overlap. Nevertheless, the functional profiles and functional redundancy indices reported here were inferred from 16S rRNA gene sequences using Tax4Fun2 and therefore represent potential functional capacities rather than directly measured gene expression or metabolic activity. Future studies integrating meta-transcriptomic profiling with targeted enzyme activity assays are needed to verify whether the predicted functions are actively expressed and translate into measurable soil processes.

Furthermore, the BC+Fe treatment enriched specific metabolic pathways (e.g., amino acid, lipid, and xenobiotics metabolism), indicating expanded nutrient utilization and detoxification capabilities that likely assist in mobilizing previously unavailable P fractions. In stark contrast, conventional BC not only reduced network complexity but also significantly increased the absolute value of negative cohesion (Figure 3a). This heightened negative cohesion was associated with P leaching loss (Figure 6a), consistent with the interpretation that P scarcity intensified microbial competition under the BC treatment. Such intense competitive interactions are detrimental to coordinated microbial functioning and constrain the expression of broader ecosystem services [42], ultimately reducing functional redundancy (Figure 7a). By physically retaining P and chemically stabilizing the soil environment, iron-modified biochar mitigated this excessive competition, maintained balanced ecological interactions, and supported a highly redundant and resilient functional structure (Figure 4b). Collectively, these results underscore the value of iron-modified biochar as a biocompatible soil amendment that harmonizes robust physicochemical P retention with the preservation of microbial network complexity.

### 4.3. P Leaching Losses and Soil pH Drive Changes in Microbial Community Structure and Network Complexity

To elucidate the mechanisms underlying the observed ecological preservation under the BC+Fe treatment, we examined the environmental drivers, specifically soil pH and P leaching loss, mediating these microbial responses. Among the variables assessed, soil pH and P leaching were most strongly correlated with microbial community composition, network complexity, and keystone species distribution. These findings align with previous studies highlighting pH and P availability as fundamental filters for bacterial assembly in agricultural soils [53,54]. Mechanistically, soil pH is known to exert direct constraints on microbial physiology by influencing membrane stability, enzyme activity, and the energy allocated for intracellular pH homeostasis, while simultaneously governing nutrient solubility and bioavailability [38,55,56].

Building on these fundamental drivers, regression analyses further underscored the cascading ecological impacts of severe P loss. Specifically, increasing P leaching was strongly associated with both the simplification of microbial interaction networks and a concomitant decline in functional redundancy (Figure 4b and Figure 6b). This relationship indicates a severe reduction in the soil’s biological buffering capacity against environmental disturbances, demonstrating that sustained P availability is non-negotiable for maintaining functional insurance in soil ecosystems. Furthermore, taxon-specific responses to P depletion highlighted divergent adaptive strategies among keystone taxa. For instance, specific *Acidobacteriota* lineages, such as *Candidatus Solibacter* (OTU1170) and *Gp1* (OTU53), are highly adapted to oligotrophic conditions, utilizing targeted phosphatase production to mineralize recalcitrant organic P when labile pools are exhausted [57,58]. Conversely, *Proteobacteria* keystone taxa, including *Mesorhizobium* (OTU86), exhibit remarkable functional versatility, employing diverse strategies—ranging from P solubilization and mineralization to intracellular storage and symbiotic associations—to sustain P cycling under fluctuating nutrient regimes [26]. The robust functional redundancy observed under the BC+Fe treatment is likely driven by the stable coexistence of these complementary microbial strategies. Crucially, P leaching loss was strongly and negatively correlated with soil pH, suggesting that physical nutrient depletion and chemical acidification act synergistically to destabilize microbial assemblages under the unmodified biochar treatment.

The intense rainfall regime used here may also have imposed physical stress on the soil matrix and thereby influenced microbial community structure and network complexity. No visible surface crusting, slumping, or collapse of the soil columns was observed during leaching; nevertheless, subtle changes in aggregate stability, pore continuity, or hydraulic conductivity cannot be excluded because these properties were not directly measured. Importantly, BC and BC+Fe received identical water inputs but differed markedly in microbial network complexity, indicating that rainfall intensity alone was insufficient to explain the stronger network simplification under BC. Rather, potential physical disturbance may have interacted with treatment-induced changes in P loss, soil pH, and microbial competition, with the latter relationships being more directly supported by our analyses.

Beyond the observed microbial and P-related responses, these findings have broader implications for sustainable agroecosystem management under increasing climatic variability and extreme rainfall events. By reducing P losses and enhancing nutrient retention, iron-modified biochar offers a dual benefit: maintaining soil fertility while lowering the risk of nutrient transfer to adjacent aquatic systems. Such dual benefits are increasingly recognized as critical components of sustainable environmental management [59,60,61]. Collectively, these considerations suggest that iron-modified biochar may represent a promising nature-based strategy that simultaneously supports soil quality, microbial functioning, nutrient conservation, and environmental protection under changing climatic conditions.

## 5. Conclusions

This study demonstrated that iron-modified biochar (BC+Fe) was more effective than unmodified biochar (BC) in mitigating P leaching from acidic soil under simulated repeated heavy rainfall. The lower P loss under BC+Fe, together with its kinetic behavior and Fe-rich surface characteristics, was consistent with enhanced Fe-associated P adsorption, while the maintenance of a higher soil pH was also associated with improved P retention. Compared with BC, BC+Fe maintained greater bacterial diversity, network complexity, and predicted functional redundancy. Reduced P leaching and weaker negative cohesion were further associated with a more interconnected microbial community and greater potential functional buffering capacity under hydrological stress.

However, these findings should nevertheless be interpreted within the limitations of the experimental design. The laboratory column system simplified natural variation in rainfall, soil structure, preferential flow, and other field conditions, while the relatively short experimental period did not capture the long-term aging and performance of iron-modified biochar. Moreover, Fe–P bonding was not directly verified spectroscopically, and Tax4Fun2-derived functional profiles represent predicted potential rather than directly measured microbial activity. Future field-scale studies integrating FTIR, XPS, or XANES characterization with metagenomic, metatranscriptomic, and enzyme activity analyses are therefore required to validate the proposed mechanisms and assess their long-term ecological relevance. Despite these limitations, iron-modified biochar shows promise as an amendment for reducing diffuse P loss while maintaining microbial structural and potential functional integrity in acidic soils exposed to intense rainfall.

## Data Availability

The microbial sequencing data obtained in this study have been submitted to National Center for Biotechnology Information (NCBI) BioProject database under accession number PRJNA1260197.

## Supplementary Materials

The following supporting information can be downloaded at: https://www.mdpi.com/article/10.3390/microorganisms14081715/s1, Figure S1: The rarefaction curves for observed OTU richness of bacterial community among different treatments; Figure S2: Energy Dispersive X-ray Spectroscopy (EDS) images of unmodified biochar (a), iron-modified biochar (b), unmodified biochar after PO_4_-P adsorption (c), and iron-modified biochar after PO_4_-P adsorption (d); Figure S3: Comparisons of relative abundance of dominant bacterial communities at the phylum level. Different letters indicate significant differences (p < 0.05) among soils; Figure S4: Comparisons of the absolute value of negative cohesion and positive cohesion of bacterial network. Different letters indicate significant differences (p < 0.05) among soils; Figure S5: Relationships between soil pH and the absolute value of negative cohesion and positive cohesion (a), and between the cumulative leaching loss of available P (LossP) and the absolute value of negative cohesion and positive cohesion (b). ns, p > 0.05; *, p < 0.05; Table S1: Physicochemical properties of apron soil across treatments.
