# Supplementary material for: Iron-Modified Biochar Reduces Phosphorus Leaching and Maintains Microbial Network Complexity in Acidic Soils Under Simulated Intense Rainfall
Source: Microorganisms. 2026 Aug 5;14(8):1715. doi: 10.3390/microorganisms14081715 (PMC13515700; doi:10.3390/microorganisms14081715)

Iron-Modified Biochar Reduces Phosphorus Leaching and Maintains Microbial Network Complexity in Acidic Soils under Simulated Intense Rainfall

Yi Luo [1], Zihao Liu [1*], Yongli Zhang [2], Chao Cui [1, 3], Geqin Wang [1, 3], Lili Dong[1], Shunli Wan[1*]

[1] College of Life and Environmental Science, Huangshan University, Huangshan 245041, China

[2] Anhui Academy of Agricultural Sciences Tea Research Institute, Hefei 230031, China

[3] College of Resource and Environment, Anhui Science and Technology University, Chuzhou 233100, China

*Corresponding author:

E-mail: wsl@hsu.edu.cn

E-mail: zhliu@hsu.edu.cn

Table S1

Table S1 Physicochemical properties of apron soil across treatments

Treatments	OM ($g\ kg^{-1}$)	TN ($g\ kg^{-1}$)	AN ($mg\ kg^{-1}$)	TP ($g\ kg^{-1}$)	AP ($mg\ kg^{-1}$)	TK ($g\ kg^{-1}$)	AK ($mg\ kg^{-1}$)	pH
CK	18.63±1.12b	1.30±0.21a	80.40±3.54a	0.03±0.00c	3.51±0.14c	25.27±0.33a	69.67±14.57c	3.9±0.2b
BC	25.53±0.42a	1.43±0.11a	82.23±18.09a	0.09±0.03b	24.39±3.29b	24.30±0.27a	95.67±3.79b	4.1±0.2b
BC+Fe	26.23±0.40a	1.31±0.28a	69.13±8.85a	0.31±0.15a	89.62±2.98a	24.57±0.19a	295.00±21.21a	4.5±0.1a

Note: Different letters indicate significant differences ($p < 0.05$).

Fig. S1 The rarefaction curves for observed OTU richness of bacterial community among different treatments.

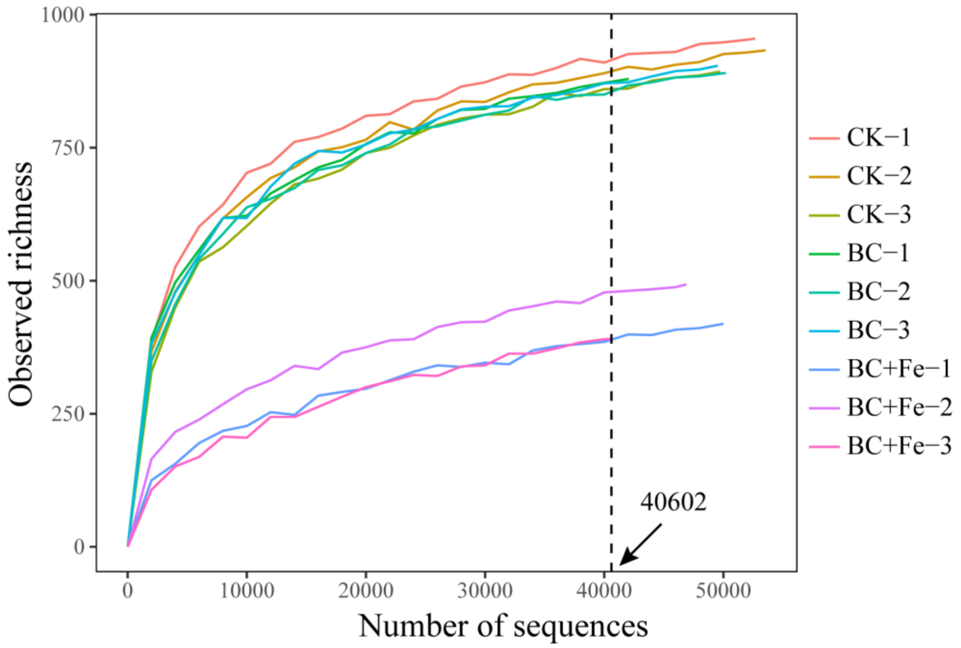

Fig. S2 Energy Dispersive X-ray Spectroscopy (EDS) images of unmodified biochar (a), iron-modified biochar (b), unmodified biochar after PO_4-P adsorption(c), and iron-modified biochar after PO_4-P adsorption (d).

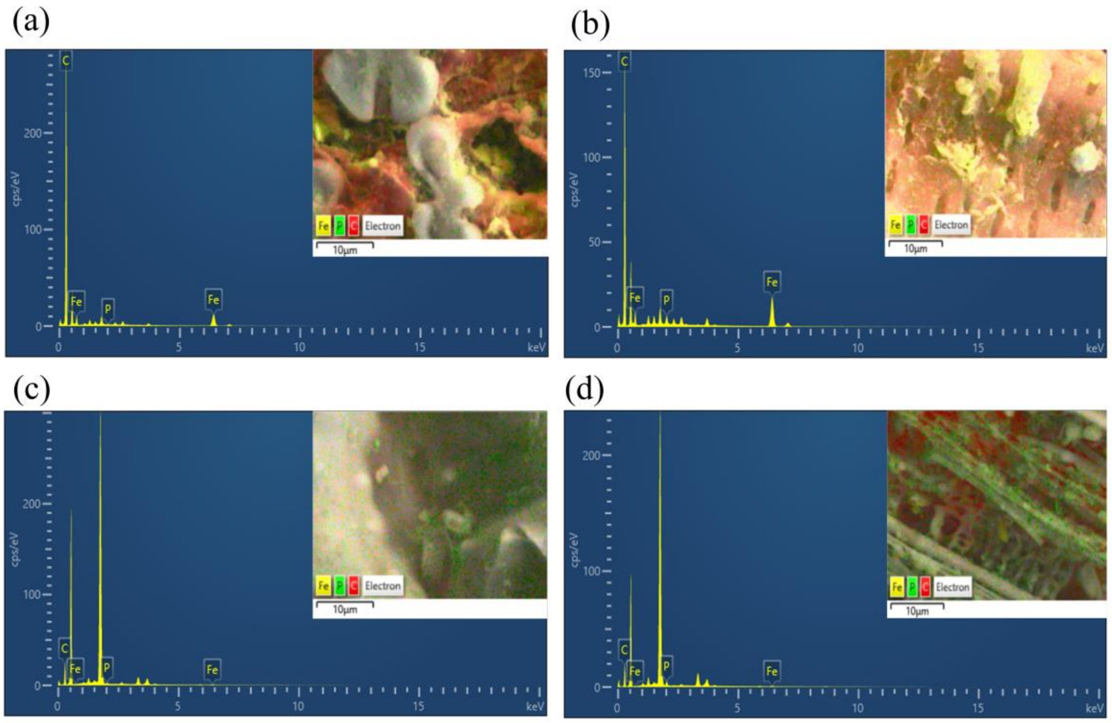

Fig. S3 Comparisons of relative abundance of dominant bacterial communities at the phylum level. Different letters indicate significant differences ($p < 0.05$) among soils.

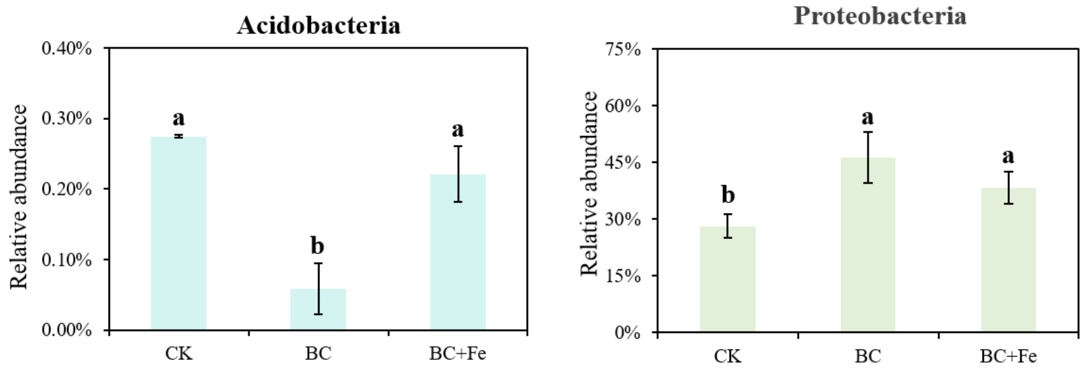

Fig. S4 Comparisons of the absolute value of negative cohesion and positive cohesion of bacterial network. Different letters indicate significant differences ($p < 0.05$) among soils.

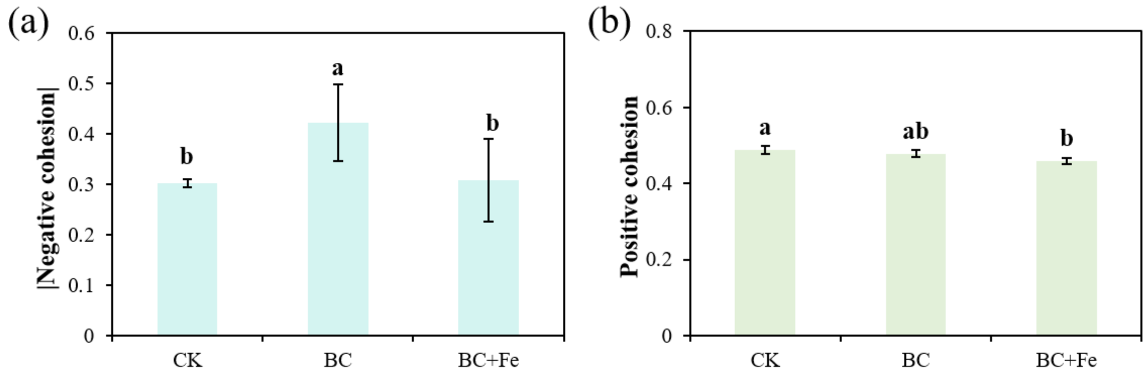

Fig. S5 Relationships between soil pH and the absolute value of negative cohesion and positive cohesion (a), and between the cumulative leaching loss of available P (LossP) and the absolute value of negative cohesion and positive cohesion (b). ns, $p > 0.05$; *, $p < 0.05$.

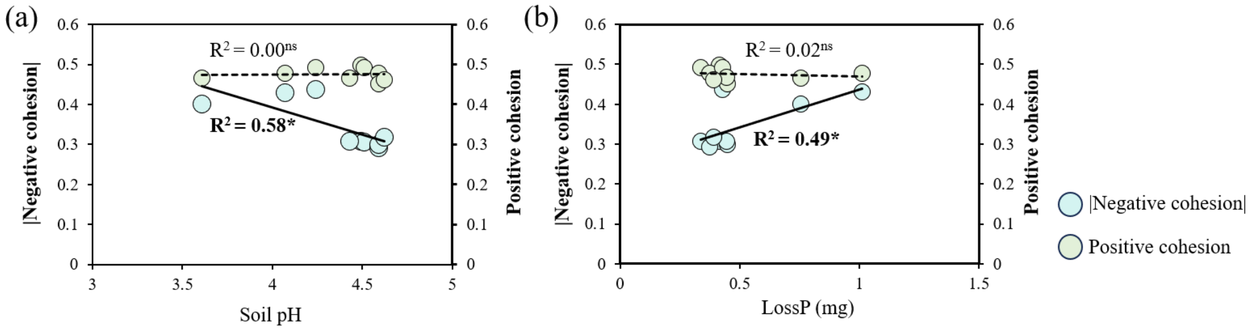

Supplement: Supplementary file 1 [file microorganisms-14-01715-s001.zip › microorganisms-4431826-supplementary.pdf]
